# Loss of TRIM67 Attenuates the Progress of Obesity-Induced Non-Alcoholic Fatty Liver Disease

**DOI:** 10.3390/ijms23137475

**Published:** 2022-07-05

**Authors:** Chao Huang, Xiaoli Wei, Qihui Luo, Yu Xia, Ting Pan, Junbo He, Asad Jahangir, Lanlan Jia, Wentao Liu, Yuanfeng Zou, Lixia Li, Hongrui Guo, Yi Geng, Zhengli Chen

**Affiliations:** 1 Laboratory of Experimental Animal Disease Model, College of Veterinary Medicine, Sichuan Agricultural University, Chengdu 611130, China; wei_xl323@163.com (X.W.); lqhbiology@163.com (Q.L.); xiayu113bvs@163.com (Y.X.); panting555666@163.com (T.P.); junbohe19@163.com (J.H.); 2019603003@stu.sicau.edu.cn (A.J.); jialanlan@sicau.edu.cn (L.J.); liuwt1986@126.com (W.L.); 2 Key Laboratory of Animal Disease and Human Health of Sichuan Province, College of Veterinary Medicine, Sichuan Agricultural University, Chengdu 611130, China; yuanfengzou@sicau.edu.cn (Y.Z.); lilixia905@163.com (L.L.); guohongrui@sicau.edu.cn (H.G.); gengyisicau@126.com (Y.G.)

**Keywords:** TRIM67, obesity, NAFLD, inflammation

## Abstract

Obesity is considered as a major cause for the development and progress of non-alcoholic fatty liver disease (NAFLD), which is one of the most prevalent chronic liver diseases worldwide. However, molecular mechanisms that implicate in obesity-driven pathophysiology of NAFLD are not well defined. Here, we report a tripartite motif (TRIM) protein family member—TRIM67—that is hardly expressed in liver but is inducible on obese conditions. Enhanced expression of TRIM67 activates hepatic inflammation to disturb lipid metabolic homeostasis and promote the progress of NAFLD induced by obesity, while the deficiency in TRIM67 is protective against these pathophysiological processes. Finally, we show that the important transcription coactivator PGC-1α implicates in the response of hepatic TRIM67 to obesity.

## 1. Introduction

An unprecedented increase in the prevalence of obesity is sweeping the globe, especially developed countries. Nearly 40% of adults are now obese in the United States, while the rate in European countries is over 20% [1]. Obesity affects nearly all physiological functions of the body and is a major cause of numerous comorbidities, such as hypertension, cardiovascular diseases, diabetes, and cancers, etc. [2,3,4,5]. Obesity is characterized by accumulated and expanded adipose tissue with chronic inflammation, which results in dysfunction of its endocrine actions. Increased secretion of adipokines, cytokines, free fatty acids and other lipid moieties by adipose tissue affects the whole-body metabolic state and promotes the development and progress of the comorbidities induced by obesity [6,7]. As the liver is one of the most important organs for lipid metabolism [8], it is not surprising that obesity is also closely correlated with the development and progress of non-alcoholic fatty liver disease (NAFLD), not only with simple steatosis, but also with nonalcoholic steatohepatitis (NASH), NASH-related fibrosis/cirrhosis, hepatocellular carcinoma and liver failure [9,10]. Besides, increased liver-specific mortality is found in obese NAFLD patients [11]. Given that accumulating hepatic lipotoxicity-mediated inflammation is thought to play a central role in NAFLD developing and progress induced by obesity [12,13], understanding the molecular mechanism of the link to obesity, hepatic lipid metabolism and immune response would provide valuable knowledge for the diagnosis and treatment of NAFLD.

Tripartite motif (TRIM) proteins are an ancient family that contains a common domain structure composed of a RING finger followed by a B-box and coiled coil domain [14]. Most of the TRIMs display E3 ubiquitin ligase activities and play important roles in diverse biological processes, such as cellular proliferation/differentiation, cell death/apoptosis, and autophagy and immune response [15,16]. More than 70 TRIM proteins were identified in humans and mice, which could be classified into subfamilies I to XI according to their different domain structure [17]. TRIM67 is a new member of the TRIM protein family, which belongs to evolutionarily conserved class I TRIMs. Previous studies have reported that TRIM67 may be implicated in the regulation of brain development [18,19]. In vitro works showed that TRIM67 would affect the progress of non-small-cell lung cancer [20,21] and colorectal cancer [22]. TRIM67 has also been shown to suppress TNFα-triggered NF-κB activation in vitro, suggesting its potential function in inflammation [23]. Nevertheless, the function of TRIM67 in vivo is not well defined. As different studies revealed that TRIM family proteins, including TRIM67, implicate in the processes of inflammation that is the key inducer of NAFLD progress, we presumed that TRIM67 may play a role in NAFLD. Thus, in this study, with TRIM67 genetically knockout mice and high-fat-diet induced obesity/NAFLD mouse models, we aim to evaluate whether the altered expression of TRIM67 could affect the development and progression of obesity-induced NAFLD and to elucidate the underlying mechanisms, which could provide new aspects for studying TRIM67 and for NAFLD treatment.

## 2. Results

### 2.1. High-Fat Diet Induces the Expression of TRIM67

**Figure 1:**
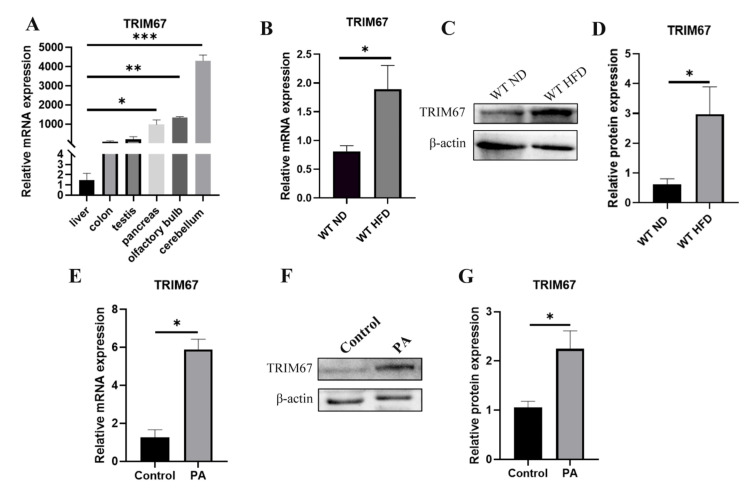
High-fat diet induces the expression of TRIM67. (**A**) Tissue expression profile of mouse TRIM67 analyzing by qRT-PCR. Error bars indicate SEM. n = 3. * *p* < 0.05, ** *p* < 0.01, *** *p* < 0.001 by one-way ANOVA. (**B**–**D**) qRT-PCR and western blots show increased hepatic TRIM67 expression in HFD-induced obesity mice. ND, normal diet. HFD, high-fat diet. Error bars indicate SEM. n = 4. * *p* < 0.05 by two-tailed Student’s *t* test. (**E**–**G**) qRT-PCR and western blots show increased hepatic TRIM67 expression in PA-treated HepG2 cells. Error bars indicate SEM. n = 3. * *p* < 0.05 by two-tailed Student’s t test.

Differently from most of the TRIMs that are ubiquitously expressed [24], TRIM67 mostly concentrates in nervous systems, especially the embryonic cortex and the adult cerebellum [18]. Our qRT-PCR data also revealed this enriched expression pattern of TRIM67 (Figure 1A). We focused on TRIM67 as we were screening the risk factors for NAFLD with animal models that we found significantly enhanced mRNA and protein levels of TRIM67 in the liver of high-fat-diet (HFD) induced obese mice (Figure 1B–D), while interestingly that the expression of which could hardly be detected in WT livers (Figure 1A). To confirm this, we used an in vitro cell model in which HepG2 cells were treated with palmitic acid (PA), finding that PA could greatly induce the expression of TRIM67 (Figure 1E–G). These results displayed the inducibility of hepatic TRIM67 upon obesity and suggested a role for TRIM67 linking obesity and liver function.

### 2.2. TRIM67 Is Not Essential for Normal Development of Mouse Liver

**Figure 2:**
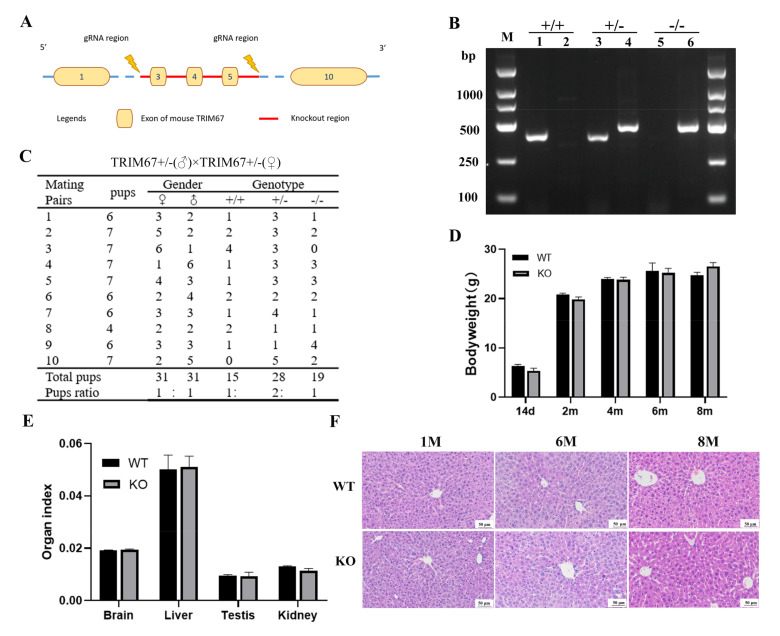
TRIM67 is not essential for normal development of mouse liver. (**A**) Overview of the targeting strategy for generating TRIM67 knockout mice. (**B**) Genotyping PCR for the validation of TRIM67 knockout mice. Wildtype allele: 391 bp; Heterozygotes: 490 bp and 391 bp; Homozygotes: 490 bp. (**C**) Quantification shows the number and frequency of offspring of each genotype produced by crossing TRIM67+/− mice. Genotypes of the pups were born with expected Mendelian frequencies. (**D**) Quantification shows normal bodyweight of TRIM67 KO mice. Error bars indicate SEM. 14 d (n = 8), 2 m (n = 8), 4 m (n = 13), 6 m (n = 4) and 8 m (n = 4). (**E**) Quantification reveals normal organ index of 18-week-old TRIM67 KO mice. Organ index indicates the ratio of organ weight to body weight. Error bars indicate SD. n = 3. (**F**) Representative images of H&E staining show normal tissue structure of TRIM67 KO liver at the age of 1 month (M), 6 M and 8 M.

To further elucidate the function of TRIM67 in the liver, we generated a TRIM67 genetically knockout mouse model (TRIM67 KO), in which the exon 3 to 5 of TRIM67 gene were deleted (Figure 2A). The genotypes of the offspring were evaluated by PCR and the KO mice were obtained by mating TRIM67 heterozygous (TRIM67 +/−) males with heterozygous females (Figure 2B). We found that TRIM67 WT (+/+), heterozygous (+/−) and KO (−/−) mice could be born with an expected Mendelian ratio (1:2:1) (Figure 2C), displaying normal postnatal viability and living for over one year. The bodyweights of adult KO mice were comparable with those of WT controls (Figure 2D), and the organ index of liver and some other important organs were also comparable between WTs and KOs (Figure 2E). Furthermore, hematoxylin-eosin (H&E) staining showed normal postnatal liver maturation of TRIM67 KO mice, of which the completed tissue structure with clear liver lobule structure and neatly arranged liver cells were observed at adult ages (Figure 2F). All these data revealed that TRIM67 is not essential for normal liver development, which could be predicted by its extremely low expression in the liver.

### 2.3. Deficiency of TRIM67 Is Protective against Hepatic Lipid Accumulation in Obese Mice

**Figure 3:**
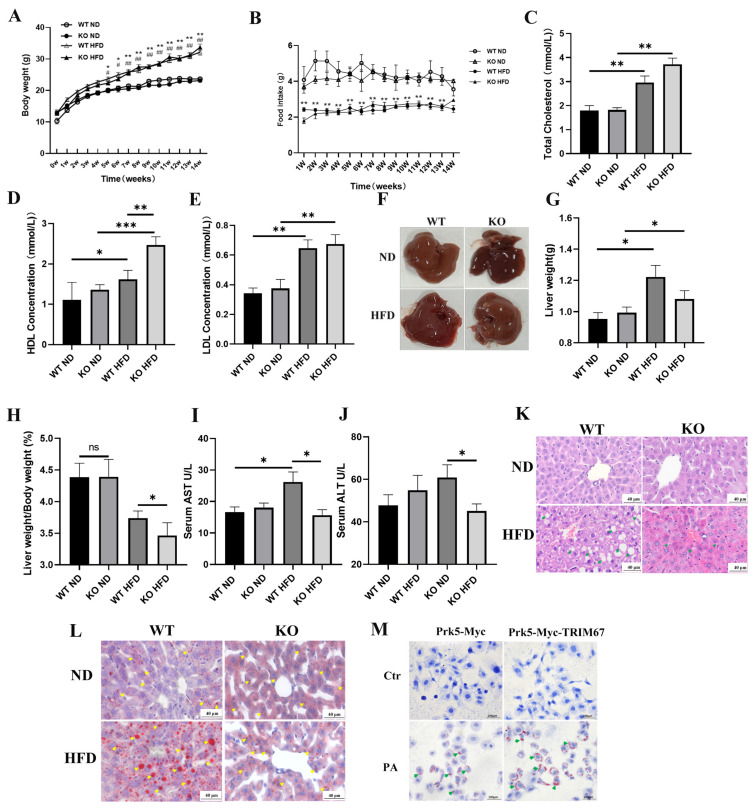
Deficiency of TRIM67 is protective against hepatic lipid accumulation in obese mice. TRIM67 WT mice and TRIM67 KO mice were fed normal diet (ND) or high-fat diet (HFD) for 14 weeks to induce obesity/NAFLD. (**A**) Sequential changes in bodyweights. Error bars indicate SD. n = 9. “*” stands for WT ND vs. WT HFD, “#” stands for KO ND vs. KO HFD. * or # *p* < 0.05, ** or ## *p* < 0.01 by two-way ANOVA. (**B**) Daily food intake of mice. Error bars indicate SEM. n = 5. * *p* < 0.05, ** *p* < 0.01 by two-way ANOVA. (**C**–**E**) Blood cholesterol, high-density lipoprotein (HDL) and low-density lipoprotein (LDL) were quantified. Error bars indicate SEM. n = 5. * *p* < 0.05, ** *p* < 0.01, *** *p* < 0.001 by one-way ANOVA. (**F**) Repetitive photographs show liver size of each group of mice. (**G**,**H**) Quantifications show greater decreased liver weight and liver index in obese TRIM67 KO mice than those in obese TRIM67 WT mice. Error bars indicate SEM. n = 4. * *p* < 0.05 by two-tailed Student’s *t* test. (**I**,**J**) Quantifications show decreased serum AST and ALT activity in obese TRIM67 KO mice compared with those in obese TRIM67 WT mice. Error bars indicate SEM. n = 4. * *p* < 0.05 by two-tailed Student’s t test (**K**) Representative images of H&E staining of liver sections show decreased hepatocellular steatosis in obese TRIM67 KO mice compared with that in obese TRIM67 WT mice. Scale bars, 20 μm. Arrows indicate hepatocellular steatosis. (**L**) Representative images of Oil Red-O staining of liver sections show greater decreased lipid accumulation in obese TRIM67 KO mice than that in obese TRIM67 WT mice. Scale bars, 20 μm. Error bars indicate SEM. n = 4. Arrows indicate accumulated lipids. (**M**) Representative images of Oil Red-O staining of PA-treated HepG2 cell. (400× magnification).

As we found increased hepatic TRIM67 expression in obese mice, we presumed the loss function of TRIM67 would be beneficial for their livers, and also be protective against the progress of obesity-induced NAFLD. Thus, we generated obesity/NAFLD mouse models through a high-fat-diet supplement, with both TRIM67 WT and KO mice. After 14 weeks of high-fat-diet supplement, TRIM67 KO mice gained comparable bodyweights to those of WT mice, and no difference in the food uptake of high-fat-diet was observed (Figure 3A,B). The levels of blood cholesterol and low-density lipoprotein (LDL) in TRIM67 KO mice displayed a comparable response to the high-fat-diet of WT mice, while a much higher high-density lipoprotein (HDL) level was observed in obese TRIM67 KO mice (Figure 3C–E). Moreover, we found that obese TRIM67 KO mice had smaller and lighter livers than obese TRIM67 WT ones (Figure 3F–H). Besides, in obese TRIM67 KO mice, we noticed a lot of normal activity of serum AST and ALT, which was significantly increased in obese TRIM67 WT mice (Figure 3I,J). As AST and ALT activity were usually used as critical indicators for liver function [25], our findings suggested that deficiency of TRIM67 would be beneficial for liver function in obese conditions. To confirm this, H&E staining was performed to evaluate this point at a histopathological level. We found observably hepatocellular steatosis in obese TRIM67 WT mice that is a typical histological feature of NAFLD, while the morphology in the liver of obese TRIM67 KO mice is histologically very normal (Figure 3K). Consistently, Oil Red-O staining that specifically stains lipid and triglyceride contents [26] showed more and larger lipid droplets in the liver of obese TRIM67 WT mice than in those of obese TRIM67 KO ones (Figure 3L). These data demonstrated that the loss of TRIM67 is protective against hepatic lipid accumulation and steatosis induced by a high-fat-diet. In support, we found that overexpression of TRIM67 could promote lipid accumulation in HepG2 cells treated with PA (Figure 3M).

### 2.4. TRIM67 KO Improves Hepatic Fibrosis Induced by High-Fat-Diet

**Figure 4:**
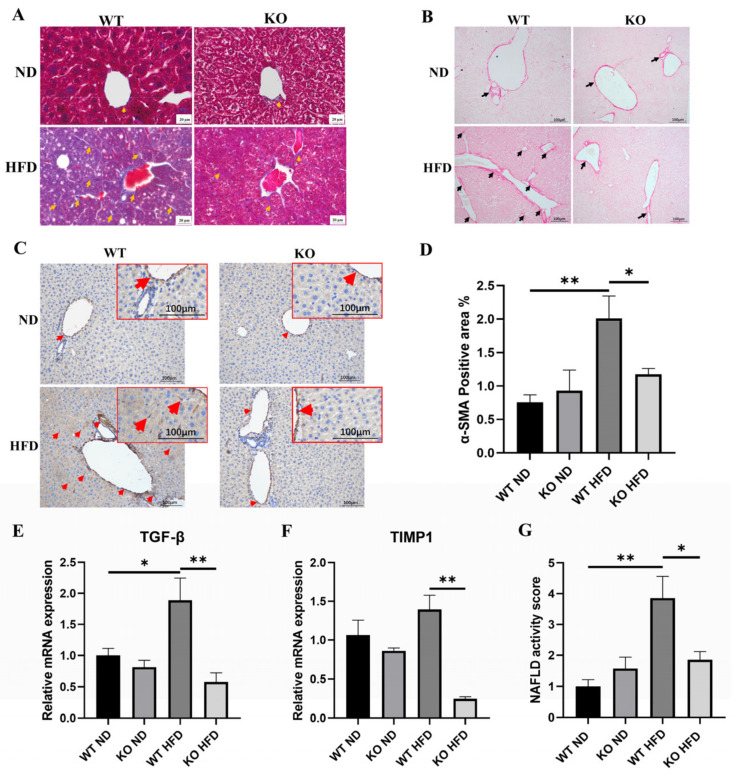
TRIM67 KO improves hepatic fibrosis induced by high-fat-diet. (**A**,**B**) Representative images of Masson staining (**A**) and Sirius Red staining (**B**) with liver sections. Scale bars, 20 μm. Errors indicate hepatic fibrosis. (**C**,**D**) Representative images of α-SMA labeling and quantification of liver sections. (200× magnification). Error bars indicate SEM. n = 3. * *p* < 0.05, ** *p* < 0.01 by one-way ANOVA. Arrows indicate α-SMA positive cells. (**E**,**F**) qRT-PCR quantifications show decreased expressions of genes related to hepatic fibrosis in obese TRIM67 KO mice compared with those in obese TRIM67 WT mice. Error bars indicate SEM. n = 4. * *p* < 0.05, ** *p* < 0.01 by one-way ANOVA. (**G**) Quantification shows decreased NAFLD activity score in obese TRIM67 KO mice compared with that in obese TRIM67 WT mice. Error bars indicate SEM. n = 6. * *p* < 0.05, ** *p* < 0.01 by one-way ANOVA.

Hepatic fibrosis is another histological feature of obesity-induced NAFLD [27]. Thus, we used Masson staining and Sirius Red staining to detect the collagen deposition in the liver, finding clearly visible signs of that in the obese TRIM67 WT liver but not in the obese TRIM67 KO liver (Figure 4A,B), which indicated an attenuated hepatic fibrosis induced by obesity after TRIM67 deficiency. Consistent with this finding, in the obese TRIM67 WT liver, we found more positive signals of α-smooth muscle-actin (α-SMA) that labels activated hepatic stellate cells (HSCs) and is a reliable marker for fibrosis [28], while many fewer of these were observed in the obese TRIM67 KO liver (Figure 4C,D). Besides, the hepatic expressions of TIMP1 and TGFβ that related to hepatic fibrosis [29] were induced by a high-fat diet in TRIM67 WT mice but not in TRIM67 KO mice (Figure 4E,F).

Since less hepatic steatosis and fibrosis indicate improved NAFLD progress, we combined the results from H&E staining and Masson staining and calculated the NAFLD activity score (NAS) [30] to evaluate the development and progress of NAFLD induced by obesity in TRIM67 WT and KO mice. We found that deficiency in TRIM67 did not affect the NAS of mice supplied with a normal diet but significantly improved the NAS of mice supplied with a high-fat-diet (Figure 4G). Collectively, these results revealed the roles of TRIM67 deficiency in protecting against obesity-induced NAFLD development and progress.

### 2.5. TRIM67 Implicates in the Regulation of Hepatic Inflammation and Lipid Homeostasis

**Figure 5:**
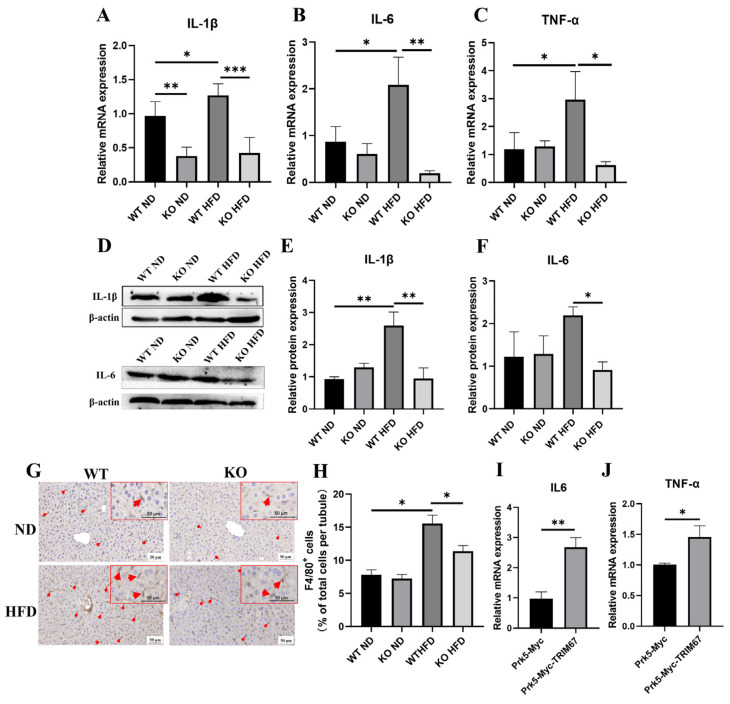
TRIM67 implicates in the regulation of hepatic inflammation. (**A**–**C**) qRT-PCR quantifications show decreased expressions of hepatic pro-inflammation factors in obese TRIM67 KO mice compared with those in obese TRIM67 WT mice. Error bars indicate SEM. n = 4. * *p* < 0.05, ** *p* < 0.01, *** *p* < 0.001 by one-way ANOVA. (**D**–**F**) Western blots and quantification show decreased protein expressions of hepatic IL-1β and IL-6 in obese TRIM67 KO mice compared with those in obese TRIM67 WT mice. Error bars indicate SEM. n = 4. * *p* < 0.05, ** *p* < 0.01 by one-way ANOVA. (**G**,**H**) Representative images and quantification of F4/80 labeling in liver of each group of mice. Error bars indicate SEM. n = 4. * *p* < 0.05, ** *p* < 0.01 by two-tailed Student’s *t* test. Arrows indicate F4/80 positive cells. (**I**,**J**) qRT-PCR shows increased pro-inflammation factors expression in HepG2 cell overexpressed TRIM67. Error bars indicate SEM. Repeated three times. * *p* < 0.05, ** *p* < 0.01 by two-tailed Student’s *t* test.

Hepatic inflammation plays a critical role in NAFLD development and progressing NAFLD from simple steatosis toward higher risk states [31]. Although complex factors are implicated in the crosstalk between hepatic inflammation and NAFLD progress, it is reported that disturbed homeostasis of hepatic lipid metabolism is a decisive way [32]. Since many TRIM family proteins play roles in inflammation [33], we presumed that TRIM67 would participate in hepatic inflammation to affect obesity-induced NAFLD progress. With qRT-PCR and western blots, we found significantly up-regulated expressions of pro-inflammation factors in the obese TRIM67 WT liver, while far fewer expressions of these were detected in the obese TRM67 KO liver (Figure 5A–F). Consistently, F4/80 labeling to display macrophage recruitment and activation [34] showed many fewer macrophages in the obese TRIM67 KO liver than in the obese TRIM67 WT liver (Figure 5G,H). These data suggested that TRIM67 is important for activating hepatic inflammation, especially in obese conditions. In support, we found that overexpression of TRIM67 could promote the expressions of pro-inflammation factors in vitro (Figure 5I,J).

**Figure 6:**
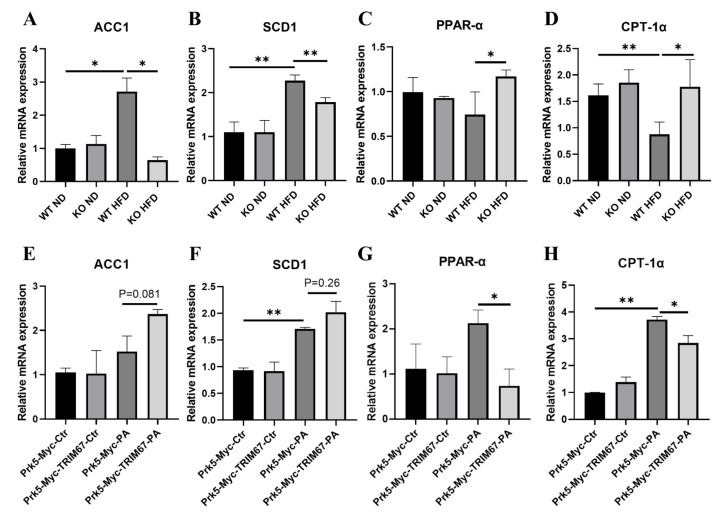
TRIM67 implicates in the regulation of hepatic inflammation and lipid homeostasis. (**A**,**B**) qRT-PCR show decreased hepatic expression of genes related with lipogenesis in obese TRIM67 KO mice compared with those in obese TRIM67 WT mice. Error bars indicate SEM. n = 3. * *p* < 0.05, ** *p* < 0.01 by two-tailed Student’s *t* test. (**C**,**D**) qRT-PCR show increased expression of genes related with fatty-acid oxidation in obese TRIM67 KO mice compared with those in obese TRIM67 WT mice. Error bars indicate SEM. n = 3 * *p* < 0.05, ** *p* < 0.01 by two-tailed Student’s *t* test. (**E**,**F**) qRT-PCR quantifications show increased expression of genes related with lipogenesis in TRIM67 overexpressed HepG2 cells treated with PA. Error bars indicate SEM. n = 3. * *p* < 0.05, ** *p* < 0.01 by two-tailed Student’s *t* test. (**G**,**H**) qRT-PCR shows decreased expression of genes related with fatty-acid oxidation in TRIM67 overexpressed HepG2 cells treated with PA. Error bars indicate SEM. n = 3. * *p* < 0.05, ** *p* < 0.01 by two-tailed Student’s *t* test.

Inflammatory signals are reported to be able to increase hepatic free fatty acid (FFA) uptake, synthase and reduce its oxidation that would initiate and promote NAFLD [32]. In congruity with this, we found that high-fat-diets induced the expressions of genes related with lipogenesis (ACC1 and SCD1) [35] in the TRIM67 WT liver, while TRIM67 deficiency significantly attenuated this induction (Figure 6A,B). In addition, the expressions of genes related with fatty-acid oxidation (PPAR-α and CPT1-α) [36] increased in the liver of obese TRIM67 KO mice, compared with those in obese TRIM67 WT mice (Figure 6C,D). Moreover, in a cultured cell model where HepG2 cells were treated with PA, we found that overexpression of TRIM67 could enhance the inducibility of ACC1/SCD1 and suppress the inducibility of PPAR-α/CPT1-α to PA insult (Figure 6E–H). All these findings revealed an essential role for TRIM67 in linking obesity and hepatic inflammation-regulated lipid metabolism.

### 2.6. PGC-1α Induces Hepatic TRIM67 in Obese Mice

**Figure 7:**
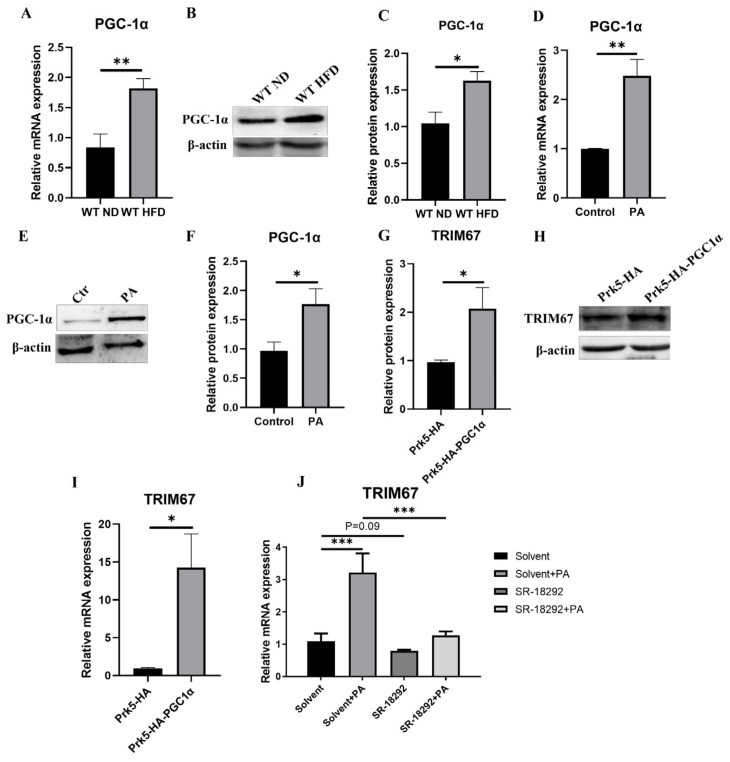
PGC-1α induces hepatic TRIM67 in obese mice. (**A**) qRT-PCR shows increased hepatic expression of PGC-1α in obese mice. Error bars indicate SEM. n = 4. ** *p* < 0.01 by two-tailed Student’s *t* test. (**B**,**C**) Western blots and quantification show increased hepatic protein expression of PGC-1α in obese mice. Error bars indicate SEM. n = 4. * *p* < 0.05 by two-tailed Student’s *t* test. (**D**) qRT-PCR shows increased expression of PGC-1α in PA-treated (600 μM, 24 h) HepG2 cells. Error bars indicate SEM. n = 3. ** *p* < 0.01 by two-tailed Student’s *t* test. (**E**,**F**) Western blots and quantification show increased protein expression of PGC-1α in PA-treated (600 μM, 24 h) HepG2 cells. Error bars indicate SEM. n = 3. * *p* < 0.05 by two-tailed Student’s *t* test. (**G**,**H**) Western blots and quantification show increased protein expression of TRIM67 in HepG2 cells overexpressed PGC-1α. Error bars indicate SEM. n = 3. * *p* < 0.05 by two-tailed Student’s *t* test. (**I**) qRT-PCR shows increased expression of TRIM67 in HepG2 cells overexpressed PGC-1α. Error bars indicate SEM. n = 3. ** *p* < 0.01 by two-tailed Student’s *t* test. (**J**) qRT-PCR shows suppression of PGC-1α activity with SR-1892 (10 µM, 24 h) decrease the inducibility of TRIM67 to PA insult. Error bars indicate SEM. n = 3. *** *p* < 0.01 by one-way ANOVA.

Peroxisome proliferator-activated receptor-γ coativator-1α (PGC-1α) interacts with multiple nuclear or non-nuclear receptors or transcriptional factors to regulate mitochondrial biogenesis, respiration, hepatic gluconeogenesis and lipid metabolism [37,38]. In our study, we noticed increased protein and mRNA levels of hepatic PGC-1α in obese mice and increased expression of that in PA-treated HepG2 cells (Figure 7A–F), which is consistent with the increased expression of TRIM67 (Figure 1B–G). Thus, we presumed that PGC-1α would implicate in the response of TRIM67 to obesity. In accordance with this hypothesis, we found that over-expression of PGC-1α can significantly promote the expression of TRIM67 (Figure 7G–I), while suppressing PGC-1α activity with its specifical inhibitor SR-18292 [39] can slightly decrease the expression of TRIM67 and greatly suppress the inducibility of TRIM67 to PA treatment in HepG2 cells (Figure 7J). These results displayed corelated implications between PGC-1α and TRIM67 in regulating hepatic function.

## 3. Discussion

In this study, we identified a novel role for TRIM67 in obesity-induced NAFLD development and progress. A high-fat-diet/obesity could promote the expression of TRIM67 to activate hepatic inflammation, accumulate hepatic lipids and progress NAFLD, while deficiency in TRIM67 is protective against these processes. Besides, in vitro experiments also reveal the inducibility of TRIM67 to PA treatment and affected expressions of genes related with lipogenesis, fatty-acid oxidation and inflammation are observed after TRIM67 overexpression. Moreover, our results further suggest a role for PGC-1α in regulating the response of TRIM67 to obesity. An interesting point is the fact that TRIM67 expresses little in mouse livers, but that elevated hepatic expression of it is present in obese mice. This expression pattern of TRIM67 and the normal hepatic development of TRIM67 KO mice reveal that hepatic TRIM67 functions not in healthy but in obese conditions, which differs from the current knowledge that TRIM67 is implicated in the normal development and function of the brain, where TRIM67 is highly expressed [15,16,37]. Except in the brain, TRIM67 expresses in some other organs; our data take us one step closer and provide new aspects in understanding the biological function of TRIM67 in vivo.

As a global health problem, overweight and obesity affected approximately 1.9 billion people and 609 million adults in 2015, respectively [40]. Besides, increased trends in the prevalence of overweight/obesity in children and adolescents have sounded the alarm [10]. Obesity is the leading cause of NAFLD, and increased prevalence of NAFLD correlates with rising trends in obesity [41]. In obese conditions, increased exposure of hepatocytes to lipids results in lipotoxicity-induced inflammation that affects the hepatic lipid metabolism and plays critical roles in developing simple liver steatosis and subsequent progressing to NASH, fibrosis and cirrhosis [42]. Thus, understanding the molecular mechanisms underlying obesity-induced NAFLD development and progress could not only be beneficial for the treatment but also for the diagnosis of NAFLD. In our study, we show that TRIM67 is a critical effector protein that links obesity and hepatic inflammation. TRIM67 KO mice display much lower hepatic inflammatory responses under obese conditions, which consequently contribute to a better balanced lipid metabolism in the liver and suppressed progress of NAFLD. These biological properties of TRIM67 are similar to those of another TRIM protein family member, TRIM8, which is also highly expressed in the liver of NASH patients and inducible to a high-fat-diet. Overexpression of TRIM8 exacerbates HFD-induced insulin resistance and gluconeogenes, while deficiency in RTIM8 ameliorates these phenotypes [43]. Besides, our data also distinguish TRIM67 from TRIM24 that is highly expressed in mouse livers. TRIM24 deficiency would result in hepatocellular lesions, steatosis, fibrosis and carcinoma, which are caused by imbalanced lipid metabolism and inflammation [44]. These findings display the implications of TRIM family proteins in hepatic function and its related disorders, and highlight the importance of TRIM67 in regulating the tissue and functional homeostasis of the liver. However, further work is needed to study the relationship between TRIM67, hepatic inflammation and lipid metabolism.

Our work also reveals a potential mechanism for TRIM67 to respond to obesity. At a transcriptional level, PGC-1α is reported to regulate the homeostasis of liver metabolism, including mitochondrial oxidative phosphorylation, gluconeogenesis, and fatty acid synthesis [45]. In obese conditions, different results are present of hepatic PGC-1α expression. Multiple studies reported that high-fat diets induced hepatic PGC-1α expression [46,47,48,49], while others showed decreased expression of that in an obese liver [50,51]. These results display the complex roles of PGC-1α in regulating liver function. In our work, we noticed enhanced PGC-1α and TRIM67 expression in the liver of obese mice and in PA-treated HepG2 cells. In vitro, overexpression of PGC-1α promotes the expression of TRIM67, while inhibition of PGC-1α activity suppresses TRIM67 expression. All of these findings display a tight correlation of PGC-1α and TRIM67 in response to obesity, and suggest a role for PGC-1α-TRIM67 axis in regulating liver function. However, more work needs to be done to elucidate specific transcriptional mechanisms for PGC-1α to regulate TRIM67, because PGC-1α does not bind DNA itself.

## 4. Methods and Materials

### 4.1. Animals

All mouse work was done in accordance with the Animal Care and Use Committee guidelines of Sichuan Agricultural University. The background of the Mouse Strains used in this study is C57BL/6N. Conventional *TRIM67* knockout mice (TRIM67−/−, KO) were generated with CRISPR-Cas9 system targeting on exon 3 to 5 of TRIM67 gene (Figure 2A) by Cyagen Biosciences (Suzhou, China). gRNA target sequence are as follows: gRNA1 (matching forward strand of gene): TCT GGG TAG GTA ACG GCT TCT GG; gRNA2 (matching reverse strand of gene): CAG GCT CAA GGG GGT CTA GAC GG. Founder mice and its offspring were identified by genotyping PCR (WT Forward: 5′-GAT GAT AGC CAT GTA ATG CCC ACC-3′, Reverse: 5′-CCG TGA TAT GCT TGC CAC AGG TTC-3′; CUT Forward: 5′-ATC AGA GAT GGA GCA GAC GCA G-3′, Reverse: 5′-TTG ATG GTT GG AGC CCT GC-3′). Genotyping was conducted using PCR analysis of genomic DNA isolated from tail clippings. All mice were bred under SPF conditions in standard individually ventilated cages at 20–22 °C, with a 12 h light/12 h dark cycle, 50–70% humidity, and ad libitum access to standard food and water.

### 4.2. Induction of Obesity/NAFLD with High-Fat Diet

Obesity/NAFLD mouse models were generated with the supplement of a high-fat diet. Briefly, 4-week-old TRIM67 WT/KO male mice were randomly divided into the normal diet group (ND, MD17131, Medicience, Yangzhou, China) and the high-fat diet group (HFD, 45% fat, MD12032, Medicience, Yangzhou, China). Then, a 14-week period supplement of indicated diets was used to induce obesity/NAFLD. The body weight and food uptake were measured weekly, and the mice were sacrificed after the treatment schedule was completed. The blood was collected and centrifuged to obtain sera and subjected to biochemical estimations (automatic biochemical analyzer, HITACHI, Tokyo, Japan).

### 4.3. Histological Staining

Livers were fixed in 4% paraformaldehyde solution and embedded in paraffin. 5 μm sections were mounted on slides and stained with hematoxylin and eosin (H&E) and Masson, according to the manufacturers’ instructions (G1120 for H&E, G1340 for Masson staining, Solarbio, Beijing, China). For Oil red O staining, frozen sections of the livers were first obtained, and the staining processes were performed according to the manufacturers’ instructions (G1260, Solarbio, Beijing, China).

### 4.4. Immunohistochemical Staining

Immunohistochemical staining was performed using a SADB-POD kit according to the manufacturers’ instructions (Boster, SA2002, Wuhan, China). Firstly, 3% H_2_O_2_ (20 min, RT) was used to block the endogenous peroxidase activity after the paraffin-embedded sections were deparaffinized and rehydrated. Secondly, the slides were washed with PBS and the antigen retrieval was performed with Citrate Buffer (pH = 6.0) under high pressure. Thirdly, the tissues sections were blocked (1 h, RT) with the blocking buffer (10% donkey serum in PBS+ 0.1% Triton X-100, if a permeabilization was needed). Then, the slides were incubated with primary antibodies (diluted in PBS with 1% donkey serum) at 4 °C overnight. The slides were washed three times with PBS and incubated with biotin labeling secondary antibody and SABC in turn (RT, 30 min). Finally, DAB was used to develop color and the slides were counterstained with hematoxylin.

### 4.5. Quantitative Realtime PCR

TRizol reagent (15596026, Invitrogen) was used to extract the total RNA from livers and cultured cells according to the previous description [52]. Then, the reverse transcription was performed with ~1 μg total RNA according to the manufacturers’ instructions (RR047A, Takara). Quantitative real-time PCR was performed with a Bio-Rad CFX96 system, and the relative gene expression was normalized to internal control as β-Actin. Primers used in this study are listed below (see Table 1).

### 4.6. Western Blots

The RIPA lysis buffer system was used to extract the proteins from liver tissues, and equal amounts of total protein from each group were separated via SDS-PAGE and transferred to polyvinylidene difluoride membranes (PVDF, Millipore, Darmstadt, Germany), followed by a 1 h block with 5% skimmed milk in TBST. Then, the membranes were incubated with primary antibodies (Table 2) at 4 °C overnight. After three washes with TBST, the membranes were incubated with secondary antibodies (1:10,000; Absin) for 1 h at room temperature, followed by three washes with TBST. Finally, protein signals were detected and scanned by the Western blot imaging system (ChemiDoc MP, Bio-rad, Hercules, CA, USA). The TRIM67 antibody was generated by immunizing rabbits with a GST fusion protein of the exon 3–5 of the mouse TRIM67 gene.

### 4.7. Cell Culture

Human hepatocellular carcinoma (HepG2) cells were obtained from the National infrastructure of cell line resource (Beijing, China). The cells were cultured in DMEM media supplemented with 10% fetal bovine serum (FBS, P30-3302, PAN, Aidenbach, Germany). To induce FFA overloading, the cells were treated with 0.6 mM palmitic acid (PA, P5585, Sigma-Aldrich, St. Louis, MO, USA) for 24 h. Control cells were incubated with the same medium containing the same amount of solvent (BSA) used to dissolve the PA. For overexpression experiments, plasmids were transfected using TransEasyTM Transfection regent (TEO-01012, Foregene, Chengdu, China). Twenty-four hours after the transfection, cells were harvested for qRT-PCR or Western blot assays. To suppress the activity of PGC-1α, 10 µM SR-18292 (SML2146, Sigma-Aldrich, St. Louis, MO, USA) was supplied into HepG2 cells for 24 h before the following assays were performed.

### 4.8. Statistical Analysis

Data represent the mean ± standard deviation (SD) or mean ± standard error of the mean (SEM). Two-tailed Student’s *t*-test or one-way ANOVA were performed for the statistical significance analysis using GraphPad Prism software (Version 6.0, San Diego, CA, USA). * *p* < 0.05, ** *p* < 0.01, *** *p* < 0.01.

## 5. Conclusions

**Figure 8:**
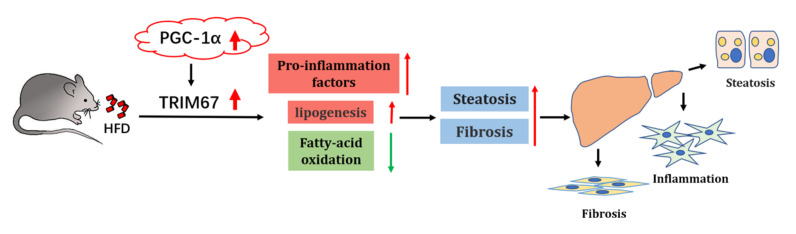
Schematic illustration of PGC-1α-TRIM67 axis mediated hepatic response to obesity. High-fat diet could induce the expression of TRIM67 with a PGC-1α dependent manner. Increased expression of TRIM67 activates hepatic inflammation to disturb hepatic lipid metabolism, resulting in increased hepatocellular steatosis and fibrosis and promoting progress of NAFLD.

In summary, this study validates a prominent role of hepatic TRIM67 in response to obese conditions to disturb the inflammatory and lipid-metabolic homeostasis of the liver, which further promotes the development and progress of NAFLD. We also show a link between PGC-1α and TRIM67 during this process, and provide evidence for TRIM67 as a diagnostic and therapeutic target for NAFLD (Figure 8).

## Figures and Tables

**Table 1:** Primers for qRT-PCR.

Gene	Primer	Sequence
Mouse *β-actin*	F	5′-AGAGGGAAATCGTGCGTGAC-3′
	R	5′-CAATAGTGATGACCTGGCCGT-3′
Mouse *TRIM67*	F	5′-GGCGAAGGAGTTTCTGGTTC-3′
	R	5′-TAGCTTCAGGGTGCAGTGATT-3′
Mouse *IL-1β*	F	5′-CCCCAGGGCATGTTAAGGAG-3′
	R	5′-TCTTGGCCGAGGACTAAGGA-3′
Mouse *IL-6*	F	5′-CTTCCATCCAGTTGCCTTCTTG-3′
	R	5′-AATTAAGCCTCCGACTTGTGAAG-3′
Mouse *TNF-α*	F	5′-ACGGCATGGATCTCAAAGAC-3′
	R	5′-GTGGGTGAGGAGCACGTAG-3′
Mouse *ACC1*	F	5′-ATTGTGGCTCAAACTGCAGGT-3′
	R	5′-GCCAATCCACTCGAAGACCA-3′
Mouse *SCD1*	F	5′-CACACCTTCCCCTTCGACTAC-3′
	R	5′-GAAACAGGAACTCAGAAGCCCA-3′
Mouse *PPAR-α*	F	5′-AACATCGAGTGTCGAATATGTGG-3′
	R	5′-CCGAATAGTTCGCCGAAAGAA-3′
Mouse *Cpt-1α*	F	5′-TGGCATCATCACTGGTGTGTT-3′
	R	5′-GTCTAGGGTCCGATTGATCTTTG-3′
Mouse *PGC-1α*	F	5′-TATGGAGTGACATAGAGTGTGCT-3′
	R	5′-CCACTTCAATCCACCCAGAAAG-3′
Mouse *α-SMA*	F	5′-CGCTGCTCCAGCTATGTGTGA-3′
	R	5′-TTTGGCCCATTCCAACCATTAC-3′
Human *ACC1*	F	5′-AGTGAGGATGGCAGCTCTGTCTC-3′
	R	5′-TGAGATGTGGGCAGCATGAAC-3′
Human *SCD1*	F	5′-GCAGGACGATATCTCTAGCT-3′
	R	5′-GTCTCCAACTTATCTCCTCCATTC-3′
Human *PPAR-α*	F	5′-CAATGCACTGGAACTGGATGA-3′
	R	5′-GTTGCTCTGCAGGTGGAGTCT-3′
Human *CPT-1α*	F	5′-CGTCTTTTGGGATCCACGATT-3′
	R	5′-TGTGCTGGATGGTGTCTGTCTC-3′
Human *PGC-1α*	F	5′-TGAAGGGTACTTTTCTGCCCC-3′
	R	5′-TCACTGCACCACTTGAGTCC-3′
Human *TRIM67*	F	5′-AAACGGACTGGACTACGAA-3′
	R	5′-ATCTGGTCCCAAACCATCTT-3′

**Table 2:** Antibodies used in this study.

Antibodies	Source	Catalog No./Dilution
IL-1β Rabbit pAb	Bioss	bs-6319R/WB 1:1000
IL-6 Rabbit pAb	ABclonal	A0286/WB 1:1000
PGC1α mouse mAb	SANTA	sc-518025/WB 1:1000
F4/80 Rabbit pAb	Bioss	bs11182R/IHC 1:500
α-SMA Rabbit mAb	HuaBio	ET1607-53/WB 1:1000 IHC 1:200

## Data Availability

Source data are provided in this paper and are available from the corresponding author upon reasonable request.
